# Multiple System Atrophy – Cerebellar Type: Clinical Picture and Treatment of an Often-Overlooked Disorder

**DOI:** 10.7759/cureus.10741

**Published:** 2020-09-30

**Authors:** Juan Fernando Ortiz, Sagari Betté, Willians Tambo, Feiyang Tao, Jazmin Carolina Cozar, Stuart Isaacson

**Affiliations:** 1 Neurology, California Institute of Behavioral Neurosciences & Psychology, Fairfield, USA; 2 Neurology, Parkinson’s Disease and Movement Disorder Center of Boca Raton, Boca Raton, USA; 3 Neurology, Universidad San Francisco de Quito, Quito, ECU; 4 Neurology, School of Medicine, University of California, Irvine, Irvine, USA; 5 Medicine, Universidad de las Américas, Quito, ECU; 6 Family Medicine, Open Door Family Medical Center, Portchester, USA; 7 Neurology, Parkinson's Disease and Movement Disorder Center of Boca Raton, Boca Raton, USA

**Keywords:** autonomic dysfunction, ataxia, parkinsonism, multiple system atrophy, sleep-wake disorder

## Abstract

Multiple system atrophy (MSA) is a rare, progressive, fatal, neurodegenerative disorder. There are two main types: the parkinsonian type (MSA-P) and cerebellar type (MSA-C). The disease usually presents with genitourinary dysfunction, orthostatic hypotension, and rapid eye movement (REM) sleep behavior disorder. Patients rapidly develop balance, speech, and coordination abnormalities. We present a review of the clinical picture and the actualized treatment modalities of the MSA cerebellar type. For the study methods, a PubMed search was done using the following medical subject headings (MeSH) terms: “multiple system atrophy/therapy". Inclusion criteria included studies in English, full papers, human studies, and publications in the last 30 years. Case reports and series were excluded. A total of 157 papers were extracted after applying the inclusion and exclusion criteria, and 41 papers were included for the discussion of this review. This review underlines the therapeutic strategies as well as the clinical picture of multiple system atrophy, and how MSA-C and MSA-P differ from each other. We discussed this review in four topics: ataxia, autonomic dysfunction (neurogenic orthostatic hypotension and urinary disorders), parkinsonism, and REM sleep disorder. In conclusion, the treatment of MSA-C is mainly symptomatic; there are not many studies on MSA-C. The ataxic component and fewer parkinsonian symptoms are the main difference of MSA-C as opposed to MSA-P.

## Introduction and background

Multiple system atrophy (MSA) is a part of the family of α-synucleinopathies. The disease is rapidly progressive and has two main types: multiple system atrophy parkinsonian type (MSA-P) and multiple system atrophy cerebellar type (MSA-C) [1]. The incidence and prevalence of this disease are 0.6 to 0.7 cases per 100,000 and 3.4 to 4.9 cases per 100,000 population, respectively [2]. The average age of diagnosis is between 55 and 60 years, and the average survival from the onset of motor symptoms is eight to nine years [3]. Cerebellar atrophy is due to olivopontocerebellar atrophy degeneration and, to a lesser extent, striatonigral degeneration (SND) [1]. Cerebellar ataxia is the main distinctive feature of the MSA-C phenotype [2].

There are not too many studies on the clinical picture of MSA-C. Most studies present MSA as a general disorder and do not differentiate between MSA-P and MSA-C. Dealing with the treatment of MSA-C is also complicated because there are not enough clinical trials involving MSA-C. The clinical picture of the disease is extensive, which is why there are only a few specifically approved treatments available, leading to frequent off-label use of medications [4]. As well, we found that MSA studies usually had small samples. For this reason, it is imperative to extend this topic, present an updated clinical picture of MSA-C, and discuss the best treatment strategies for dealing with the numerous problems of this disease.

We aim to consolidate the knowledge of this atypical disorder and present the treatment of MSA-C, which may differ from MSA-P. We will conduct a thorough literature review to show the main clinical features of MSA-C and focus on evidence to differentiate it from MSA-P before presenting the updated treatment strategies available for MSA-C to enhance awareness of this overlooked disorder, which also requires complex treatments.

## Review

Methods

A search strategy was implemented using medical subject headings (MeSH) and regular keywords for data collection studies in PubMed and Google Scholar with the following inclusion and exclusion criteria.

Inclusion Criteria

1. Human subjects

2. Papers published in English

3. Observational studies, clinical trials, cross-sectional studies, or reviews

4. All full papers

Exclusion Criteria

1. Studies on animals

2. Non-English language studies

3. Case series, case reports, or editorials

We present four sections in the discussion below: ataxia, parkinsonism, autonomic dysfunction (neurogenic orthostatic hypotension and urinary disorders), and rapid eye movement (REM) sleep disorder. We briefly present important insights into these disorders and then discuss possible treatment strategies in each of the symptoms of MSA-C. In our study, we identified 12 clinical trials.

Results

After the application of our criteria, a total of 157 articles were selected (Table 1), of which 109 were eliminated for one of the following reasons: case report, case series, meta-analysis, systematic reviews, or lack of outcome of the study. Forty-eight publications were used to conduct this literature review.

**Table 1:** The number of articles found after applying inclusion/exclusion criteria MeSH: Medical subject headings.

MeSH keyword/subheading "multiple system atrophy/therapy"	
Total records	466
Inclusion/exclusion criteria	
Humans	428
English language	348
Published within 30 years	321
Full-text paper	157

Discussion

MSA-C is an uncommon disorder, as we detailed previously. Treating MSA-C and differentiating from MSA-P are a clinical challenge for physicians. Considering this, we decided to review the main clinical features of MSA-C and discuss how it is differentiated from MSA-P. We also present an updated treatment by symptoms because it has no cure and is only treated symptomatically.

Ataxia and Cerebellar Pathology

Cerebellar symptoms are seen in 100% of patients with MSA-C compared to 54% of MSA-P patients [5]. Sometimes, MSA-C can be challenging to differentiate from other similar disorders such as idiopathic late-onset cerebellar ataxia (ILOCA) [6] and sporadic adult-onset ataxia (SAOA) of unknown etiology. Although these entities share common symptoms with MSA-C, both are exclusion diagnoses and are distinguished as acquired ataxia rather than hereditary, and even more than that, they lack parkinsonism and orthostatic symptoms, which are key findings of MSA-C [7]. In MSA-C, cerebellar dysfunction manifests as gait ataxia, limb ataxia, ataxic dysarthria, and eye movement abnormalities such as dysmetria, saccadic intrusion, and ocular dysmetria [8].

Spinocerebellar ataxias (SCAs) are a group of autosomal dominant cerebellar ataxia (ADCA) characterized by several aspects across families, such as the age of onset, symptoms, and clinical evolution. There are many different types of SCAs, depending on the mutated gene. According to clinical and pathologic characteristics, ADCAs are classified into types I to III. ADCAs I and II present some of the same symptoms - progressive cerebellar ataxia, supranuclear ophthalmoplegia, pyramidal and extrapyramidal signs, moderate dementia, optical atrophy, and peripheral neuropathy - whereas ADCA II also manifests as macular and retinal degeneration. ADCA III consists of pure late-onset cerebellar ataxias [9]. Table 2 compares the characteristics of ILOCA, SAOA, and SCAs, and Table 3 shows the comparison between MSA-P and MSA-C.

**Table 2:** Chart comparison of ILOCA, SAOA, and SCAs ILOCA: Idiopathic late-onset cerebellar ataxia; SAOA: sporadic adult-onset ataxia of unknown etiology; SCAs: spinocerebellar ataxias (SCAs); MSA-C: multiple system atrophy – cerebellar type.

	ILOCA	SAOA	SCAs
Heritability	Idiopathic/ not defined	Non-hereditary	Mostly autosomal dominant
Progression	Slow progression	Slow progression	Slow progression
Survival	Normal life span	Unknown	Variable, there is a wide range of 68-80 years depending on the type of mutation.
Onset	41.1 ± 14.2 years	Starting around the age of 50 years	Variable, different mutations in distinct loci are associated with this variation.
Neurogenic orthostatic hypotension	Rarely occurs	Seen in more than 50% of patients as a part of mild autonomic dysfunction	Nonspecific information found
Urinary incontinence	Appears in the late phase of the disease but with less frequency than MSA-C	More than 50% of patients have it as a part of mild autonomic dysfunction.	Presents as a part of the symptoms; however, urinary frequency was the most common symptom followed by voiding difficulty.
Extrapyramidal dysfunctions	Rare	Cerebellar ataxia	Yes, in combination with pyramidal symptoms

**Table 3:** Chart comparison between MSA-P and MSA-C MSA-P: Multiple system atrophy – parkinsonism type; MSA-C: multiple system atrophy – cerebellar type.

	MSA-P	MSA-C
Heritability	Sporadic	
Progression	Rapid deterioration	
Mean survival	9 ± 4 years	8 ± 3 years
Onset	Age 56 ± 10	Definite MSA-C 54.7 ± 8.5 years; possible/probable MSA-C 56.8 ± 8.7 years
Neurogenic orthostatic hypotension	Common in both phenotypes, usually emerges after urogenital symptoms	
Urinary incontinence	Common at the time of first evaluation	
Clinical characteristics	Mainly rigidity, bradykinesia, postural instability, and/or tremor; anhidrosis more common; more severe, and widespread cognitive dysfunctions	Mainly gait ataxia, limb ataxia, dysarthria, and/or nystagmus; anhidrosis less common; less severe visuospatial and constructional dysfunctions
MRI findings	Hypointensity of the posterior putamen, hot cross bun sign, hyperintense T2 signal in the shape of a cross within the pons it is produced from degeneration of transverse pontocerebellar fibers (both MSA-C and MSA-P)	More frequent abnormalities, especially cerebellar/pons atrophy, hyperintensities of the middle cerebellar peduncles, hot cross bun sign, hyperintense T2 signal in the shape of a cross within the pons it is produced from degeneration of transverse pontocerebellar fiber (both MSA-C and MSA-P)

Treatment 

Currently, only symptomatic treatment is available, which includes pharmacologic and nonpharmacologic therapies. We focused on pharmacology approaches, which have been organized according to the main neurotransmitter involved in each symptom.

1. Glutamate antagonist: Riluzole is a glutamate receptor antagonist that decreases the toxicity of glutamate on neurons. A clinical trial by Seppi et al. showed that a dose of 200 mg was well-tolerated but failed to prove efficacy on extrapyramidal symptoms in patients with MSA [10]. Nevertheless, it was noted that they used the unified Parkinson's disease rating scale (UPDRS), which does not reflect the clinical picture of MSA due to the mixture of cerebellar and parkinsonian phenotypes. A direct study of riluzole therapy and its impact on the cerebellar symptoms of MSA have not been described. However, another clinical trial of riluzole among patients with hereditary ataxias (Friedreich ataxia or spinocerebellar ataxia) showed a potential benefit [11]. The drug seems to be safe and well-tolerated and improved the scale for the assessment and rating of ataxia (SARA) score. The proportion with the decreased SARA score was 14 (50%) in 28 patients in the riluzole group in contrast to three (11%) of 27 in the placebo group.

2. Gamma-aminobutyric acid (GABA) analog: A total of 10 patients with cortical cerebellar atrophy showed improvement in an open study after receiving a single dose of 400 mg gabapentin as well as daily administration of 900 to 1600 mg of gabapentin in four weeks. The lack of GABAergic neurons in the cerebellar cortex reduces Purkinje cell signals, resulting in disorders such as ataxia. Gabapentin counteracted those anomalies and then showed promising improvement in these disorders, highlighting the importance of GABA pathways in normal brain activities [12].

3. Serotonin agonist: Tandospirone is a serotonin (5HT-1) receptor partial agonist similar to buspirone. In a clinical study, 39 patients were given 15 mg per day of tandospirone [13]. Among the 39 patients, 12 had MSA (five MSA-C and seven MSA-P). Clinical improvement was not observed in these patients, but there was an improvement in patients with Machado-Joseph disease (MJD) and spinocerebellar atrophy 6 (SCA6). A possible explanation of why tandospirone was most effective in these patients is that serotonin pathway enhancement may exert an inhibitory effect on the glutamate release on Purkinje cells, subsequently triggering more modulations in neuron output.

Parkinsonism

Patients with MSA tend to show a lack of response to carbidopa-levodopa and have a more rapidly progressive disease than Parkinson's [14]. Because of the lack of response to carbidopa-levodopa, other strategies have been developed to treat this disease such as dopamine agonists and amantadine, but they were found no more effective than levodopa (L-Dopa) [15]. As expected, parkinsonian symptoms are more common in MSA-P than MSA-C (seen in 97.6% and 73.5% of patients, respectively), according to a cohort developed in the United States [16].

Treatment

1. Dopamine: Two relevant cohorts with a significant number of patients have been published on carbidopa-levodopa combination, one in Europe and the other in the United States [5,16]. In Table 4, we see the main results of those cohorts. 

**Table 4:** Summary of clinical response rate and duration of carbidopa-levodopa in patients with MSA in the European and American cohort studies MSA-C: Multiple system atrophy cerebellar type; MSA-P: multiple system atrophy parkinsonian type; L-Dopa: levodopa.

	Response to L-Dopa in MSA-C patients	Response to L-Dopa in MSA-P patients	Response duration (years) in MSA-C patients	Response duration (years) in MSA-P patients	Total number of cases
European cohort study [5]	13%	31.2%	3.5	3.3	141
American cohort study [16]	25%	25%	2.6	3.3	126

As we see in the results above, MSA-P patients utilized more dopamine agonists than MSA-C patients. Because patients with MSA-P have more parkinsonian symptoms, they were expected to use dopamine agonists more frequently. In both studies, MSA-P had a better response to L-Dopa than MSA-C [5,16].

Side effects are usually more common among atypical parkinsonism than in Parkinson's disease (PD). A study of 34 patients, 10 of them with MSA, showed that 90% had side effects compared to 23.2% of patients with PD [17]. There was no reasonable explanation for different responses to treatment and the higher incidence of side effects in patients with MSA than PD [17].

A recent clinical trial involving 61 patients with atypical parkinsonian syndromes utilized the dopamine agonist rotigotine (RTG), which is a non-ergoline drug and showed promising results. During the study, the UPDRS-III, mini-mental state examination (MMSE) and adverse events (AEs) were recorded. Overall, UPDRS-III scores improved. As for the mental status, there was no correlation between medication use and MMSE. Adverse effects were reported in 26% of patients, and only 11.4% of the patients dropped out. The author argues that because of the sustained effect of the drug (24 hours deliver patch), the dopamine in these patients remains stable, and the activation of D3 receptors compensates hypodopaminergic function [18]. Regardless of the positive results, more information needed to be conducted about the efficacy of rotigotine.

2. Glutamate antagonist: Despite amantadine being an antiviral agent, it has been discovered with other targets, such as glutamate and acetylcholine (ACh). Specifically, amantadine is an N-methyl-D-aspartate (NMDA) receptor antagonist that decreases glutamate levels and enhances dopamine activity in the brain improving movement disorder in Parkinson’s disease. A small clinical study of eight patients showed that a 200 mg dose of amantadine showed a tendency of reduction in the UPDRS-III scores [19]. Nevertheless, the results were not significant, for the subscores of rigidity, akinesia, tremor, postural instability, and gait disorder failed to reach significance. A recent study of parenteral amantadine showed good tolerability and efficacy in treating MSA-P [20]. The researchers used a more appropriate measure: the unified multiple system atrophy rating scale (UMSARS), instead of the UPDRS, as a means to prove efficacy. However, the study was not double-blind, and the sample size was small. Although there are no specific studies on amantadine for MSA-C, it could be applied considering the evidence in PD and MSA-P treatment.

Autonomic Dysfunction

Autonomic dysfunctions are very common in MSA and have been related to secondary damage and loss of neurons in the intermediolateral part of the spinal cord and loss of catecholaminergic neurons in the ventrolateral medulla [21]. Autonomic dysfunction is a hallmark of both types of MSA, and there is not a clear difference between them [1].

I. Neurogenic Orthostatic Hypotension

Early, severe neurogenic orthostatic hypotension (nOH) is a presenting symptom that manifests with postural lightheadedness, dizziness, sensation of blacking out, and falls with or without syncope [22]. There are medications that can successfully improve orthostatic hypotension. Nonetheless, drugs such as midodrine have been shown to worsen supine hypertension [23].

Treatment

1. Alpha/beta agonist: Droxidopa is an oral noradrenaline precursor approved by the Food and Drug Administration (FDA) in 2014. On a molecular level, droxidopa is an artificial amino acid precursor, which is converted centrally and peripherally to norepinephrine. In a phase III study among patients with PD, MSA, pure autonomic failure, or diabetic autonomic neuropathy, droxidopa was well-tolerated and proved to increase standing systolic blood pressure [24]. Four of six nOH symptoms of a severity scale significantly improved, namely dizziness, lightheadedness, fainting, or blackout feelings. Besides the improvement of symptoms, patients improved daily activities. A randomized withdrawal study of patients with symptomatic neurogenic orthostatic hypotension was conducted in treatment responders [25]. Although the study failed to meet statistical significance, it still showed a favorable trend.

2. Acetylcholine enhancement: Pyridostigmine inhibits acetylcholinesterase activity and increases ACh in the synaptic cleft. Pyridostigmine amplifies sympathetic ganglionic neurotransmission. In theory, it should increase baroreceptor-reflex proportionate to the orthostatic changes, which would elevate standing hypotension without worsening supine hypertension as it occurs with other medications [26-28]. A clinical study with 60 mg of pyridostigmine bromide showed an increase in standing blood pressure one hour after administration. There was no correlation between the drug and post-drug noradrenaline levels [23].

3. Alpha-1 agonist and corticoid: Midodrine and fludrocortisone are the oldest drugs studied in this review. The FDA approved midodrine in 1996. Midodrine is a prodrug that undergoes enzymatic hydrolysis to become an active metabolite. The drug does not cross the blood-brain barrier like other sympathomimetic drugs such as ephedrine or amphetamines. Midodrine acts peripherally as an alpha-1 adrenergic agonist [29]. There are no specific clinical trials or observational studies of MSA and midodrine. Nevertheless, three clinical trials were conducted in patients with autonomic failure due to different conditions: PD, MSA, and pure autonomic failure. Midodrine was well-tolerated overall in all studies. We reviewed the main findings of the three clinical trials in Table 5 [29-31]. Because supine hypertension can be worsened with medications, it is recommended to withhold midodrine at the end of the day or if the patient is going to be recumbent [29].

**Table 5:** Summary of clinical trials of midodrine in patients with neurogenic orthostatic hypotension (nOH) nOH: Neurogenic orthostatic hypotension; PD: Parkinson's disease; MSA: multiple system atrophy.

Author/date	Number of subjects	Number of subjects with MSA	Study type and method	Study outcome
Jankovic et al., 1993 [30]	97	18	A double-blind, placebo-controlled study in patients with autonomic failure and one of the following conditions: pure autonomic failure, MSA, and PD	Efficacious in moderate-to-severe orthostatic hypotension associated with autonomic failure. Subgroup efficacy was not studied. But the Parkinson’s patients showed the most improvement.
Wright et al., 1998 [29]	27	7	A double-blind, placebo-controlled, four-way cross-over trial with patients with autonomic failure, multiple system atrophy, and Parkinson’s disease.	10 mg of midodrine, three times a day, was effective for standing systolic hypotension. There was a significant linear relationship between midodrine dosage and mean systolic blood pressure. Subgroup efficacy was not studied.
Low et al., 1997 [31]	171	16	A multicenter, randomized, placebo-controlled study in patients with pure autonomic failure, MSA, PD, diabetes mellitus, and other diseases.	A dose of 10 mg, three times a day was efficacious and safe in the treatment of nOH. There was an increase in the standing systolic blood pressure and improvement in the global symptom relief score.

Fludrocortisone is a mineralocorticoid with weak glucocorticoid activity. Studies of fludrocortisone have not been made on MSA; still, there is evidence of efficacy in PD with the use of 0.1-0.4 mg per day [32]. Fludrocortisone is usually a second-line therapy due to side effects including hypercortisolism. 

II. Erectile Dysfunction

Erectile dysfunction (ED) is usually an early manifestation of MSA and a symptom of autonomic failure. Beck et al.'s study showed the ED prevalence of 96% in male MSA patients and as the first symptom in 37% [33]. Specific data regarding MSA-C was not found.

Treatment

Nitric oxide (NO): A cross-over study of 24 patients, 12 of them with MSA, showed an improvement of ED with sildenafil. However, orthostatic hypotension was a problematic side effect that prompted the discontinuation of the medication in three patients. Treating ED improved quality of life in patients with MSA-C, but orthostatic hypotension is a potential complication. Sildenafil acts as an inhibitor of cyclic guanosine monophosphate (cGMP)-specific phosphodiesterase (PDE)-5. Penile erection involves relaxation of the corpus cavernosum by release of NO and cGMP. The blood pressure can fall > 30 mm Hg, which, in addition to orthostatic hypotension, can result in fatal outcomes. Providers will have to evaluate the risks and benefits of prescribing this medication for this group of patients given its rapid onset of side effects in one hour [34,35].

III. Urinary Incontinence

Urge incontinence, nocturia, and incomplete neurogenic bladder have been described in MSA. Urge incontinence in patients with MSA-C had a prevalence of 68.5% and 82% in the European and American studies, respectively, while MSA-P had a prevalence of 75% and 89% in the same studies [5,16].

Treatment

Specific studies on MSA and urinary incontinence have not yet been performed or found in our research, but there is sufficient evidence to treat these common conditions. Urge incontinence is caused by detrusor hyperreflexia, and sphincter detrusor dyssynergia may be relieved by the use of anticholinergics such as oxybutynin (22.5 mg/day). Of course, anticholinergics carry the risk of cognitive decline. Botulinum injection also has shown promising results. Nocturia could be alleviated by desmopressin (5 mg intranasal at night). Finally, regarding incomplete bladder emptying, alpha-adrenergic and intermittent catheterization are treatment options that prevent recurrent urinary infections [36].

Pharmacological therapy is usually first line for ED, yet other alternatives, such as testosterone or alprostadil injections, are usually recommended to patients because of the side effects of sildenafil. Urinary problems are treated empirically in MSA-C patients because of the lack of specific studies.

Sleep Disorders

Sleep disorders usually precede classical motor signs of MSA. They include REM sleep behavior disorder (RBD), nocturnal stridor, and insomnia. The body is usually atonic during REM sleep, but this response is not the case in RBD, and patients can act out their dreams [37]. In terms of prevalence, a meta-analysis of 64 patients by Palma et al. reported the following findings: Signs of RBD were present in 78% of patients with MSA-C and 86% with MSA-P [38]. Giannini et al. found that RBD was the first symptom in 27% of patients, preceding the classic clinical features with a median of 3 (2-5) years [37].

Treatment

There are no observational studies or clinical trials specific for MSA, but commonly used drugs to treat RBD are melatonin, clonazepam, and cholinergic agents.

1. Melatonin and GABA enhancement: This is usually a first-line choice treatment. It maximizes the atonia during REM sleep and is better tolerated than clonazepam, another first-line treatment for RBD. A small study of 14 patients, two of them with MSA, reported improvement with and without concomitant use of clonazepam [39]. A low dose of clonazepam (0.5 to 1 mg) has also been effective in treating RBD. Clonazepam is a benzodiazepine that increases GABA activity. There are some side effects of clonazepam including sexual dysfunction and cognitive impairment, which make clonazepam less desirable. Currently, there are no specific studies on clonazepam therapy for RBD in MSA patients [40].

2. Acetylcholine: Rivastigmine increases ACh levels by inhibiting acetylcholinesterase. There are no studies on rivastigmine and MSA, but a double-blind cross-over trial of 12 Parkinson’s patients found a reduced average frequency of RBD episodes in patients who failed previous therapies [41]. The cholinergic side effects of rivastigmine such as nausea, vomiting, diarrhea, and headaches make the drug less desirable than the previous options mentioned above [41].

Clonazepam is a drug with more proved efficacy, but because of its adverse effects, we may start with a melatonin and use clonazepam when melatonin therapy has failed. More research on rivastigmine needed to be made. For now, rivastigmine could be used as an off-label therapeutic option whenever other medications have failed in MSA-C.

## Conclusions

Cerebellar symptoms, including gait ataxia, limb ataxia, dysarthria, and nystagmus, are the hallmarks of MSA-C and seen in half of the MSA-P cases. They can be challenging to differentiate from other medical conditions, and only limited symptomatic management is available. Direct evidence of ataxia treatment for MSA lacks, but a glutamate antagonist riluzole, a GABA analog gabapentin, and a serotonin agonist tandospirone are potential candidates for further research. As expected, MSA-C has fewer parkinsonian symptoms than MSA-P. MSA-P overall has a better response to levodopa and the other dopamine agonist than MSA-C. Nevertheless, the response of MSA of both types is inferior and much lower than Parkinson’s disease. The glutamate antagonist amantadine needs more evidence to prove efficacy for both types of MSA; in the meanwhile, amantadine could be considered a second-line drug therapy. 

Autonomic dysfunction is a hallmark of both MSA types, and there is no apparent clinical difference between them. Droxidopa and pyridostigmine are the more modern drugs of nOH. Droxidopa has more evidence of efficacy in comparison to pyridostigmine. Even so, pyridostigmine does not cause supine hypertension. The older drug midodrine and fludrocortisone continue to be used but are less desirable. Midodrine increases supine hypertension, and fludrocortisone causes hypercortisolism, which is unhelpful. The enhancement of the alpha one receptor on the vascular system is the main target for droxidopa and midodrine treatment. Fludrocortisone enhances catecholamine activity. In contrast, pyridostigmine amplifies sympathetic ganglionic neurotransmission of the baroreceptor-reflex in proportional to the orthostatic changes. Sleep disorders are a prodromal symptom in MSA-C. Melatonin is the first line of treatment, followed by GABA enhancement (Clonazepam). Increasing the levels of ACh with rivastigmine is a second-line solution for patients who failed previous therapies.
